# Trace metal bioaccumulation in oysters (*Crassostrea gigas*) from Liaodong Bay (Bohai Sea, China)

**DOI:** 10.1007/s11356-020-11968-6

**Published:** 2021-01-06

**Authors:** Yonghua Liu, Jiayu Xu, Yong Wang, Song Yang

**Affiliations:** College of Animal Husbandry and Veterinary, Jinzhou Medical University, No. 40 Songpo Street Linghe District, Jinzhou, 121000 Liaoning Province China

**Keywords:** Trace metal, Bioaccumulation, Oyster, Season, Size, Liaodong Bay

## Abstract

Cd, Cr, Cu, Pb, and Zn concentrations were measured in oysters (*C. gigas*), plankton, and seawater during spring, summer, and autumn in Liaodong Bay (Bohai Sea, China) to elucidate the effects of season, region, and oyster size on metal bioaccumulation in oysters. Metal concentrations were quantified via atomic absorption spectrophotometry. Our study determined that metal concentrations in oysters, plankton, and seawater were the highest in summer, whereas the lowest levels occurred in autumn. Regarding oyster sizes, the highest Pb levels occurred in C3-sized oysters (> 5-cm length), whereas the highest Cd, Cr, Cu, and Zn levels occurred in C2 (3–5-cm length) oysters. In contrast, the lowest Cu and Pb levels occurred in C1 (< 3-cm length) oysters, whereas the lowest mean Cd, Cr, and Zn concentrations were observed in C3 oysters. Significant differences in trace metal concentrations in the three sample types were observed in all sampling sites.

**Supplementary Information:**

The online version contains supplementary material available at 10.1007/s11356-020-11968-6.

## Introduction

With the development of human activities, various anthropogenic pollutants have been continuously discharged into the marine environment. Trace metals are considered the most important kind of marine pollutants (Rabaoui et al. 2020, and 2014; Aydin and Tokalioglu 2015; Zhou et al. 2014; Le et al. 2016). Due to their persistence, non-degradability, bioaccumulation, and toxicity, trace metals can not only pollute seawater and sediments but can also be accumulated in marine organisms through the food chain and become a human health threat when consumed (Shenai-Tirodkar et al. 2016; Rabaoui et al. 2017; Wang et al. 2013; Yaman et al. 2014).

In recent decades, serious environmental pollution has resulted from the rapid economic and industrial development of China’s coastal areas. A large amount of trace metals such as cadmium (Cd), chromium (Cr), copper (Cu), lead (Pb), and zinc (Zn) are released into the marine environment, all of which bioaccumulate in seafood and pose a serious human health risk (Li et al. 2018; Zhang et al. 2016; Gao et al. 2019). Liaodong Bay is a semi-enclosed water body located in the northern region of the Bohai Sea, which is impacted by trace metal pollution derived from several industrial and agricultural activities along its coast. Therefore, monitoring trace metal pollution in Liaodong Bay is crucial (Zhang et al. 2016; Eroglu et al. 2015).

A number of marine organisms have been used as bioindicators of trace metal pollution in marine habitats, including bivalves, gastropods, and cephalopods (Liu et al. 2017; Wang et al. 2005; Rabaoui et al. 2017; Thébault et al. 2008). Oysters are filter-feeding bivalves that are common in marine environments. These organisms are known to accumulate trace metals and are thus often used as bioindicators for trace metal monitoring in marine ecosystems (Melwani et al. 2014; Lu et al. 2020). Oysters have a high nutritional value and are thus consumed in large quantities by humans. However, oysters containing high levels of trace metals are dangerous for consumers. Therefore, determining trace metal concentrations in oysters is of the utmost importance (Lee et al. 2015; Weng and Wang 2019).

Previous studies on the accumulation of trace metals were mostly concentrated in the Bohai Bay and Laizhou Bay of the Bohai Sea; however, only few studies have been conducted in Liaodong Bay. In this study, the concentrations of Cd, Cr, Cu, Pb, and Zn were determined in oysters *Crassostrea gigas* (*C. gigas*), plankton, and seawater collected from five sampling sites in Liaodong Bay. The main objectives of this study were (1) to determine trace metal burdens in oysters from Liaodong Bay; (2) to clarify the effect of season, region, and oyster size on trace metal accumulation in oysters from Liaodong Bay; and (3) to evaluate whether the trace metal levels in oysters from Liaodong Bay adhere to safety thresholds and guidelines.

## Materials and methods

### Sample collection and analyses

**Fig. 1:**
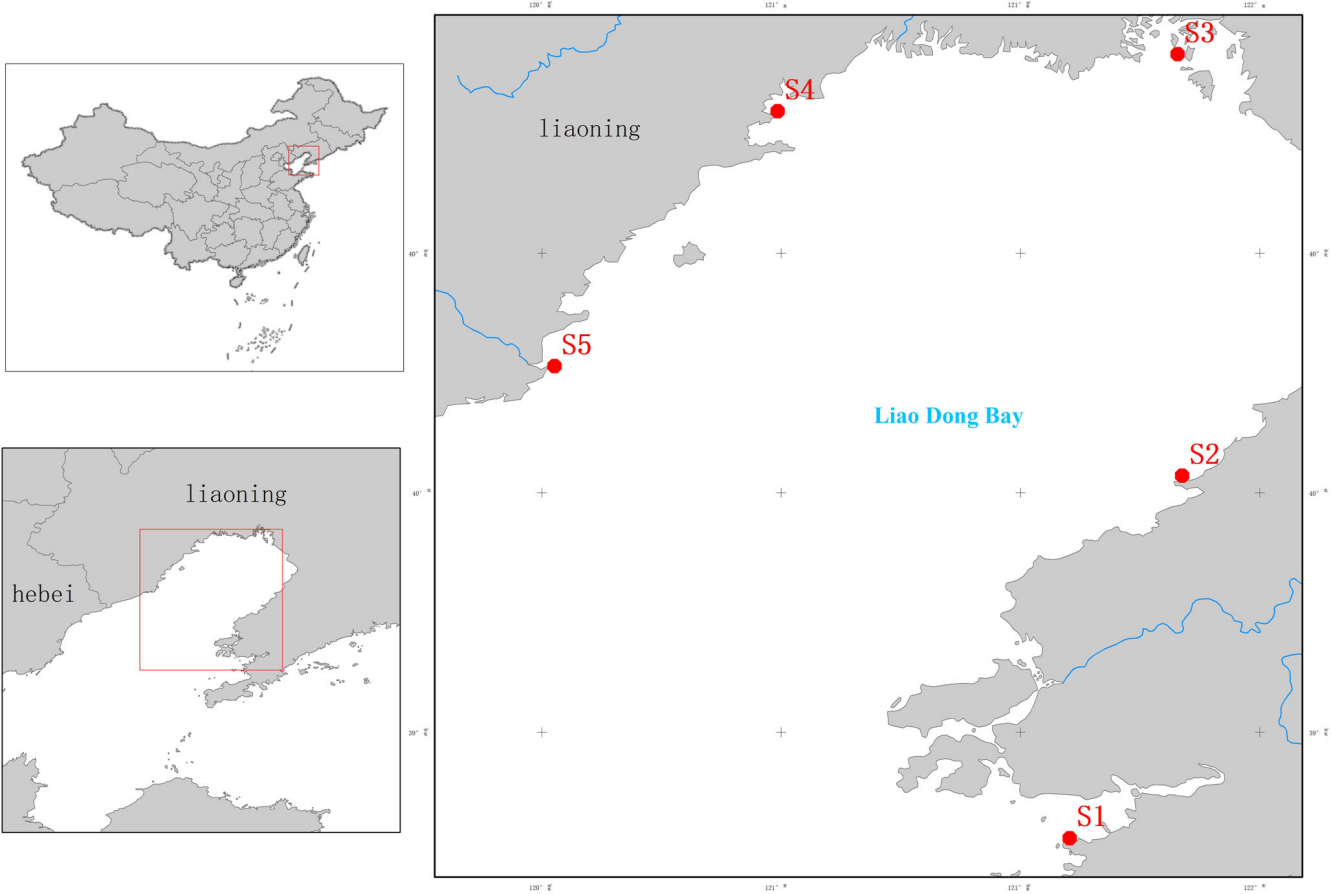
Sampling sites in Liaodong Bay. S1, Daling; S2, Bayuquan; S3, Panjin; S4, Jinzhou; S5, Suizong

Sampling was conducted from spring to autumn (2017) in Liaodong Bay, Bohai Sea, China (Fig. 1). *C. gigas*, a common local species, was collected from sampling sites along Liaodong Bay. The oysters were transferred to the laboratory on ice and then stored at − 20 °C until required (Liu et al. 2017).

The length and weight of *C. gigas* were measured and recorded. Oysters were classified into three categories based on length (C1: < 3 cm, C2: 3–5 cm, C3: > 5 cm) (Shakouri and Gheytasi 2018).

The soft tissues of 3–5 oysters were separated and homogenized to obtain 18–20 individual samples from each site, and regional information was averaged. The samples were then dried until they reached a constant weight, and wet and dry weights were recorded for each sample (Lu et al. 2017). Chemical digestion was then performed. Briefly, each 1 g sample was digested with 7 ml nitric acid (65%) and 3 ml of cholic acid (AR, Sinopharm Chemical Reagent Co., Ltd., China). The samples were predigested at 40 °C for 1 h and then digested at 140 °C for 4 h (Shakouri and Gheytasi 2018). The samples were then cooled, diluted with double distilled water, and filtered. Analysis of trace metals was carried out using an Agilent 220FS atomic absorption spectrophotometer (Agilent Technologies, Santa Clara, CA, USA).

Seawater was collected from a < 1-m depth and stored at 4 °C until analyzed (Shakouri and Gheytasi 2018). Moreover, a 100-μm mesh net was used to collect plankton from a < 10-m depth. The plankton samples were mixed with 10 ml ethanol (96%) in bottles and stored at − 20 °C. The samples were then thawed and filtered and then dried and weighed. Afterward, the samples were chemically digested using 6 ml nitric acid (65%) (Yap et al. 2002; Shakouri and Gheytasi 2018) and analyzed in the using an Agilent 220FS atomic absorption spectrophotometer following the guidelines of the Marine Monitoring Specifications (GB 17387.4-2007a; GB 17387.6-2007b). The analytical methods used herein are summarized in Supplementary Table S1.

In order to ensure accuracy, standard reference material (1566b oyster tissue) and replicate analyses were conducted for quality control. Quality control samples were repeatedly measured every 20 samples. The recoveries were within 89% and 110% of the reference values.

### Data analyses

All data were analyzed using the SPSS 18.0 software (IBM; Armonk, NY, USA). Analysis of variance (ANOVA) was conducted to compare the means, after which the Student-Newman-Keuls test was conducted to identify differences between means. Differences between the means of different groups of samples were considered statistically significant at *P* < 0.05. Each value was expressed as the mean ± standard deviation.

## Results

### Trace metal concentrations in *C. gigas*

**Fig. 2:**
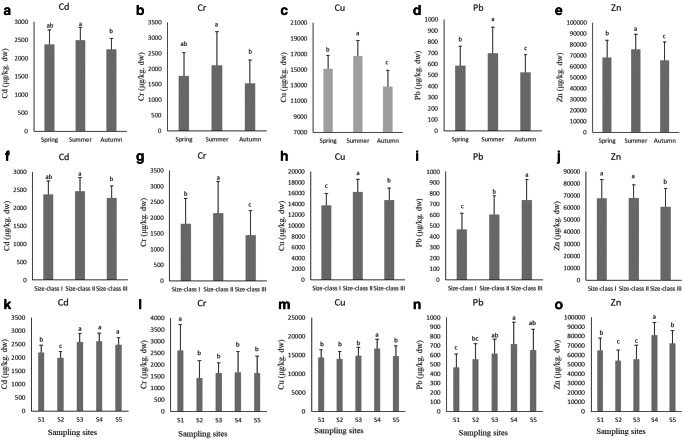
Trace metal concentrations in *C. gigas* soft tissues at different seasons, sizes, and sampling sites. Different letters indicate significant differences (*P* < 0.05, *n* = 18–20). **a**–**e** represent the seasonal changes in trace metal concentrations in oysters; **f**–**j** represent the changes in trace metal concentrations in oysters as a function of oyster size; **k**–**o** represent the changes in trace metal concentrations in oysters in different sampling sites

The trace metal concentrations in *C. gigas* varied significantly (*P* < 0.05) depending on season, size, and sampling site. Figure 2 illustrates these changes in *C. gigas* trace metal concentrations. Mean trace metal concentrations in oysters were the highest in summer and the lowest in autumn (*P* < 0.05, A-E in Fig. 2).

Regarding oyster size, the lowest mean Cu and Pb concentrations occurred in C1-sized oysters, whereas the lowest mean Cd, Cr, and Zn concentration means occurred in C3 oysters. Conversely, the highest Pb mean occurred in C3 oysters, whereas the highest Cd, Cr, Cu, and Zn means occurred in C2 oysters (*P* < 0.05, F-J in Fig. 2).

In terms of oyster sampling sites, the highest Cd means occurred in sites S3, S4, and S5, and the lowest Cd means occurred in sites S1. The Cr means were higher in site S1, and the Cu means were higher in site S4 compared to the other sites. The highest Pb means occurred in site S4, and the lowest Pb means occurred in site S1. The highest Zn means occurred in site S4, and the lowest Zn means occurred in sites S2 and S3 (*P* < 0.05, K-O in Fig. 2). Table 1 compares the heavy metal concentrations in oysters from different regions.

**Table 1:** Comparison between trace metal concentrations in oysters from the Liaodong Bay and other areas (mg/kg)

Site	Species		Year	Cd	Cr	Cu	Pb	Zn	Reference
Liaodong Bay	*Crassostrea gigas*	d. w.	Spring, 2017	2.38	1.78	15.30	0.58	68.23	This study
			Summer, 2017	2.50	2.11	16.76	0.70	75.57	
			Fall, 2017	2.24	1.53	12.85	0.53	65.58	
Chinese coastal waters	*Crassostrea gigas*, *Crassostrea ariakensis*, *Crassostrea* spp., *Crassostrea sikamea*, *Crassostrea nippona*, etc.	d. w.	2015	7.66	10.65	522	0.92	2068	Lu et al. (2017)
Xiamen in China	Oyster (no specified species)	w. w.	2013–2014	0.10–0.14	ND-0.80	–	0.01–0.06	–	Zhao et al. (2016)
Mazatlan Bay	*Crassostrea iridescens*	d. w.	2001	2.30	0.99	86.90	2.30	1161	Soto-Jimenez et al. (2001)
The southern coast of Korea	Crassostrea gigas	w. w.	2009–2013	0.59	0.22	32.48	0.15	154.38	Mok et al. (2015)
Goa, Central-West Coast of India	Oyster	d. w.	2013–2014	7.10–88.50	–	134.40–2167.90	0.10–1.70	–	Shenai-Tirodkar et al. (2016)
Sonora, Mexico	*Crassostrea corteziensis*	w. w.	Winter, 2004	0.90	–	3.11	0.53	–	García-Rico et al. (2010)
			Spring, 2005	3.24	–	2.73	0.64	–	
			Summer, 2005	4.92	–	3.45	0.67	–	
			Fall, 2005	3.85	–	1.72	0.39	–	
Chabahar bay	*Saccostrea cucullata*	d. w.	Spring, 2018	2.00	–	6.39	1.75	15.20	Shakouri and Gheytasi (2018)
			Summer, 2018	2.19	–	6.77	2.07	21.08	
			Fall, 2018	1.70	–	5.90	1.67	10.28	

### Trace metal concentrations in plankton

**Fig. 3:**
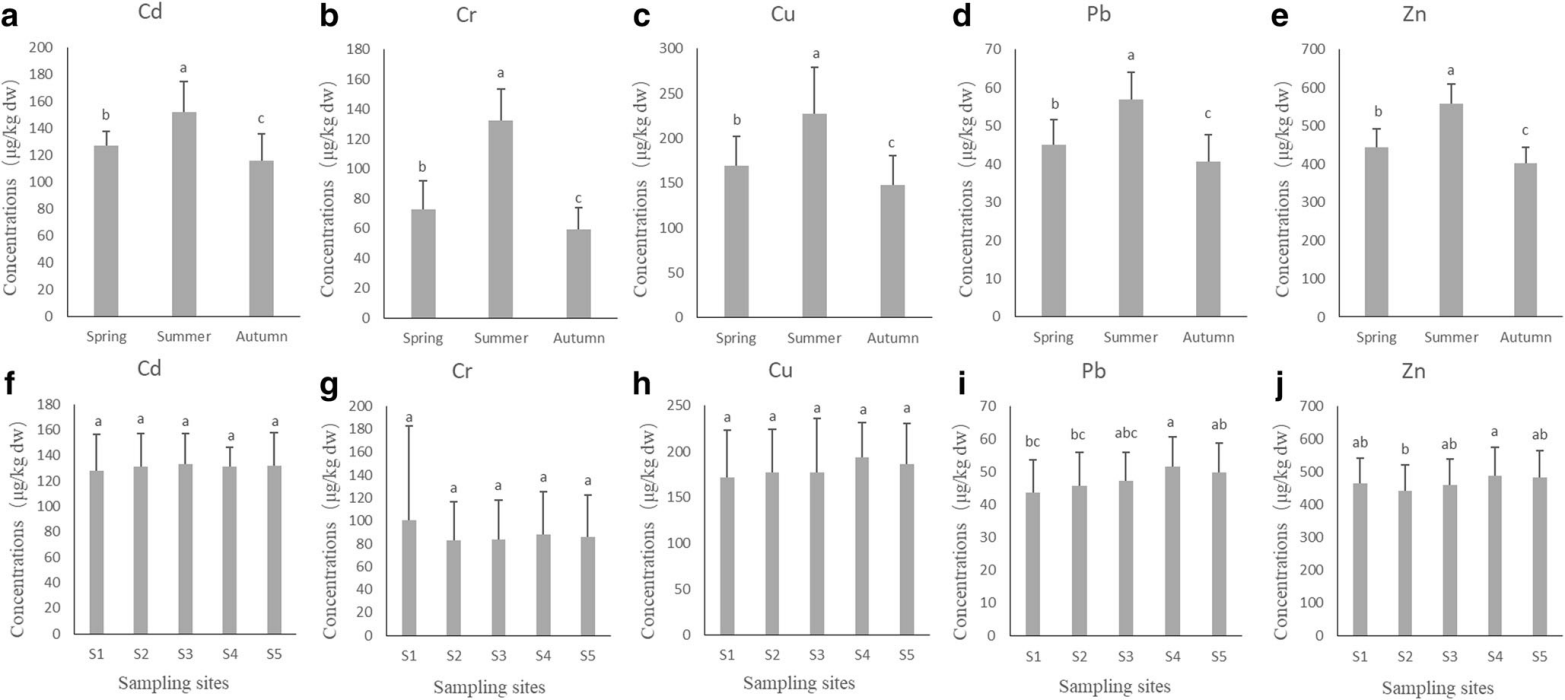
Trace metal concentrations in plankton samples in different seasons and sampling sites. Different letters indicate significant differences (*P* < 0.05, *n* = 15). **a**–**e** represent the seasonal changes in trace metal concentrations in plankton; **f**–**j** represent the changes in trace metal concentrations in plankton in different sampling sites

Trace metal concentrations in plankton are illustrated in Fig. 3. The seasonal order of trace metal concentrations in plankton was as follows: summer > spring > autumn (*P* < 0.05, A-E in Fig. 3). The mean Pb concentrations in plankton were the highest in S4. The lowest Pb levels occurred in sites S1 and S2. The mean Zn concentrations in plankton were the highest in site S4. The lowest Zn levels occurred in site S2 (*P* < 0.05, F-J in Fig. 3). No significant differences were observed between the mean Cd, Cr, and Cu concentrations.

### Trace metal concentrations in seawater

**Fig. 4:**
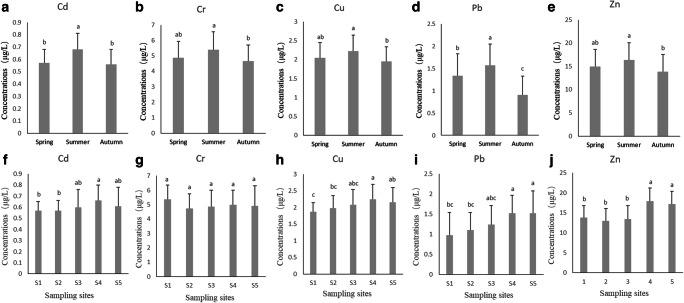
Trace metal concentrations in seawater samples in different sites and sampling sites. Different letters represent significant differences (*P* < 0.05, *n* = 15). **a**–**e** represent the seasonal changes in trace metal concentrations in seawater; **f**–**j** represent the changes in trace metal concentrations in seawater in different sampling sites

Trace metal concentrations in seawater are illustrated in Fig. 4. Seawater trace metal concentrations were higher in summer than in spring and autumn (*P* < 0.05, A-E in Fig. 4). The Cd means were higher in S3, S4, and S5 than in S1 and S2. The Cu means were higher in S4 than in S2; the Pd means were higher in S4 and S5 than in S1 and S2; and the Zn means were higher in S4 and S5 than in S1, S2, and S3 (*P* < 0.05, F-J in Fig. 4). Cr concentrations did not vary significantly between sampling sites.

## Discussion

### Seasonal variation

In this study, the trace metal concentrations of oysters, plankton, and seawater were higher in summer than in autumn, which may be because plankton growth is more accentuated in the summer. Given that oysters feed on plankton, the higher abundance of plankton during the summer season may lead to higher trace metal accumulation rates in oyster tissues (Shakouri and Gheytasi 2018). High river inputs in summer bring more metal pollution, which may be another reason for the seasonal variations in metal accumulation in marine ecosystems (Lu et al. 2020; Weng and Wang 2019). Similar seasonal variations in trace metal concentrations in oysters were also found in Sepetiba Bay (Brazil), as well as in Culiacan, Mazatlan, and Sonora (Mexico) (Rebelo et al. 2003; Soto-Jimenez et al. 2001; García-Rico et al. 2010).

### Size-dependent variation

Richards and Chaloupka (2008) found that metal accumulation in oysters was independent of size. Moreover, Yesudhason et al. (2013) found that oysters of the same size were not necessarily the same age and weight. Due to the irregular shape of the materials that attach to oysters, these organisms tend to grow along different axes. Additionally, there is enough space inside the oyster shell for these organisms to develop properly regardless of the outer shell size or shape. However, our study determined that the accumulation of metals in oysters was size-dependent. The metal concentrations in C2 oysters were higher than those in C1. This result is consistent with the observations of Shakouri and Gheytasi (2018). Interestingly, the lowest mean Cr, Cd, and Zn concentrations occurred in C3 oysters, suggesting that oysters may have different metal accumulation capacities as they grow.

### Spatial variation

Trace metal concentrations in the three sample types investigated herein were found to exhibit significant differences between sampling sites. Except for Cr, all trace metal concentrations were high in S4. This result may be attributable to anthropogenic activities in the study area, such as shipyards, chemical plants, and zinc-copper mines. Although industrial wastewater must be purified before being discharged, a large amount of metal pollutants within the permissible discharge concentrations range are still discharged into the Liaodong Bay each year. Liaodong Bay possesses a weak water exchange capacity, and therefore pollutants cannot be quickly transported to open water. Therefore, metal pollutants tend to accumulate in seawater and aquatic organisms (Liu et al. 2017; Gao et al. 2013; Zhang et al. 2018). Trace metal concentrations were low in the S1 and S2 sites, which may be due to the lower occurrence of industrial complexes in these locations.

### Nutritional standard evaluation

Our results were also considered from a dietary exposure perspective. The Cd concentrations in *C. gigas* exceeded the national safety guidelines of the People’s Republic of China (GB 2762-2017) in all of the sampling sites studied herein. Although the concentrations of other trace metals were within the GB 2762-2017 standards, our results demonstrate that Cd pollution in oysters from Liaodong Bay poses a substantial human health risk and thus should be monitored closely.

The results of this study indicate that the bioaccumulation of trace metals in oysters from Liaodong Bay was season-, spatial-, and size-dependent. In the future, the distribution of trace metals in organisms of different trophic levels will be studied to further clarify the spatial and temporal distribution of trace metals, monitor trace metal pollution, and provide a basis for the control of trace metal pollution in Liaodong Bay. The Cd concentration of oysters in the study area was relatively high, and therefore future research should focus on the Cd-associated health risks of seafood consumption in this region.

## Conclusions

Our study quantified trace metal concentrations in oysters, plankton, and seawater in Liaodong Bay to characterize oyster trace metal bioaccumulation in this region. The trace metal concentrations in oysters, plankton, and seawater varied by season, site, and oyster size. Trace metal concentrations in oysters, plankton, and seawater were the highest in summer, whereas the lowest levels occurred in autumn. Regarding oyster size, the highest levels of Pb occurred in C3; and the highest levels of Cd, Cr, Cu, and Zn occurred in C2. Conversely, the lowest levels of Cu and Pb occurred in C1, whereas the lowest Cd, Cr, and Zn occurred in C3. Significant site-dependent differences in trace metal concentrations were observed in the three sample types studied herein.

## Supplementary information

ESM 1 (DOCX 15 kb)

## Data Availability

The datasets supporting the results of this article are included within the article and associated files.
