# Supplementary material for: Trace metal bioaccumulation in oysters (Crassostrea gigas) from Liaodong Bay (Bohai Sea, China)
Source: Environ Sci Pollut Res Int. 2021 Jan 6;28(16):20682–9. doi: 10.1007/s11356-020-11968-6 (PMC8099804; doi:10.1007/s11356-020-11968-6)
Supplement: Supplementary file 1 — (DOCX 15 kb) [file 11356_2020_11968_MOESM1_ESM.docx]

Supplementary Table S1 Analytical techniques and detection limits.

| Detection limit | Cd | Cr | Cu | Pb | Zn |
| --- | --- | --- | --- | --- | --- |
| Seawater (μg/L) | 0.01 | 0.4 | 0.5 | 0.02 | 3.1 |
| Organism (μg/kg) | 5 | 40 | 400 | 40 | 400 |

Note: All trace metals were analyzed via flameless atomic absorption spectroscopy except for Zn, which was analyzed via flame atomic absorption spectroscopy.
